# Characterization of upper airway microbiome across severity of COVID-19 during hospitalization and treatment

**DOI:** 10.3389/fcimb.2023.1205401

**Published:** 2023-07-04

**Authors:** Lowell Ling, Christopher K.C. Lai, Grace Lui, Apple Chung Man Yeung, Hiu Ching Chan, Chung Hon Shawn Cheuk, Adonia Nicole Cheung, Lok Ching Chang, Lok Ching Sandra Chiu, Jack Zhenhe Zhang, Wai-Tat Wong, David S. C. Hui, Chun Kwok Wong, Paul K. S. Chan, Zigui Chen

**Affiliations:** ^1^ Department of Anaesthesia and Intensive Care, Faculty of Medicine, The Chinese University of Hong Kong, Hong Kong, Hong Kong SAR, China; ^2^ Department of Microbiology, Faculty of Medicine, The Chinese University of Hong Kong, Hong Kong, Hong Kong SAR, China; ^3^ Department of Medicine and Therapeutics, Faculty of Medicine, The Chinese University of Hong Kong, Hong Kong, Hong Kong SAR, China; ^4^ Faculty of Medicine, The Chinese University of Hong Kong, Hong Kong, Hong Kong SAR, China; ^5^ Stanley Ho Centre for Emerging Infectious Diseases, Faculty of Medicine, The Chinese University of Hong Kong, Hong Kong, Hong Kong SAR, China; ^6^ Department of Chemical Pathology, Faculty of Medicine, The Chinese University of Hong Kong, Hong Kong, Hong Kong SAR, China

**Keywords:** COVID-19, SARS-CoV-2, 16S rRNA, upper airway microbiome, intensive care unit

## Abstract

Longitudinal studies on upper respiratory tract microbiome in coronavirus disease 2019 (COVID-19) without potential confounders such as antimicrobial therapy are limited. The objective of this study is to assess for longitudinal changes in the upper respiratory microbiome, its association with disease severity, and potential confounders in adult hospitalized patients with COVID-19. Serial nasopharyngeal and throat swabs (NPSTSs) were taken for 16S rRNA gene amplicon sequencing from adults hospitalized for COVID-19. Alpha and beta diversity was assessed between different groups. Principal coordinate analysis was used to assess beta diversity between groups. Linear discriminant analysis was used to identify discriminative bacterial taxa in NPSTS taken early during hospitalization on need for intensive care unit (ICU) admission. A total of 314 NPSTS samples from 197 subjects (asymptomatic = 14, mild/moderate = 106, and severe/critical = 51 patients with COVID-19; non–COVID-19 mechanically ventilated ICU patients = 11; and healthy volunteers = 15) were sequenced. Among all covariates, antibiotic treatment had the largest effect on upper airway microbiota. When samples taken after antibiotics were excluded, alpha diversity (Shannon, Simpson, richness, and evenness) was similar across severity of COVID-19, whereas beta diversity (weighted GUniFrac and Bray–Curtis distance) remained different. Thirteen bacterial genera from NPSTS taken within the first week of hospitalization were associated with a need for ICU admission (area under the receiver operating characteristic curve, 0.96; 95% CI, 0.91–0.99). Longitudinal analysis showed that the upper respiratory microbiota alpha and beta diversity was unchanged during hospitalization in the absence of antimicrobial therapy.

## 1 Introduction

Coronavirus disease 2019 (COVID-19) is a respiratory illness caused by a novel coronavirus (SARS-CoV-2) (Huang et al., 2020). The clinical presentation of patients with COVID-19 varies from asymptomatic to critical, which can result in severe pneumonia, acute respiratory distress syndrome, multi-organ failure, and death (Lui et al., 2020). Age and baseline comorbidities such as renal failure, cardiovascular disease, and obesity have been established as the major risk factors of severe COVID-19 infection (Dessie and Zewotir, 2021). Host genetic variants are also associated with COVID-19 mortality (Pairo-Castineira et al., 2021). Although the current omicron variant is much weaker than the original variants of SARS-CoV-2, understanding of host–virus interaction remains incomplete (Esper et al., 2022).

Recent insight into the role of microbiome in human disease has opened up potential new therapeutic avenues (Sorbara and Pamer, 2022). Once thought to be sterile, the dynamic microbiome in the lung has only been recently recognized (Hilty et al., 2010). Furthermore, asthma, cystic fibrosis, and pneumonia are associated with changes in lung microbiome different to that of healthy lungs (Dickson et al., 2016a; Goldman et al., 2018). It remains controversial whether altered microbiome is the result of lung disease, contributes to the disease process itself, or both (Dickson et al., 2014). Nevertheless, modifying the lung microbiota with probiotics has already shown promise in reducing exacerbations in cystic fibrosis (Weiss et al., 2010).

Because SARS-CoV-2 is primarily a respiratory infection, upper airway respiratory dysbiosis-inflammation may play a role in determining severity of COVID-19. Indeed, it has been shown that the respiratory tract microbiome is different in patients with COVID-19 compared with healthy ones (Mostafa et al., 2020; Xu et al., 2020; Rhoades et al., 2021). Furthermore, some studies have shown that COVID-19 severity is associated with progressive changes in upper airway respiratory microbiota (Merenstein et al., 2021; Shilts et al., 2021; Ventero et al., 2021). However, current evidence is often conflicting, likely due to small sample sizes and differences in cohort selection, sampling time points, and site of sampling (Mostafa et al., 2020; Braun et al., 2021; Llorens-Rico et al., 2021; Shilts et al., 2021; Wu et al., 2021; Merenstein et al., 2022). In addition, many studies did not report or account for antimicrobial use in patients hospitalized for COVID-19 (Ma et al., 2021; Merenstein et al., 2021; Rueca et al., 2021; Ventero et al., 2021; Chen et al., 2022). As up to 75% of patients with COVID-19 were given antimicrobial therapy early on during the pandemic, this may have affected the respiratory microbiome independent of SARS-CoV-2 infection (Langford et al., 2021). Last, longitudinal studies of dynamic changes in COVID-19 respiratory microbiome are scarce (Llorens-Rico et al., 2021; Merenstein et al., 2021; Ren et al., 2021; Xu et al., 2021; Candel et al., 2023). We hypothesized that changes in upper respiratory microbiota in hospitalized patients with COVID-19 over the course of hospitalization are greatly affected by treatments such as antimicrobial therapy. Nevertheless, as COVID-19 is associated with respiratory inflammation, we postulate that upper respiratory microbiota may be related to COVID-19 severity, viral load, and plasma cytokines. The primary objective of this prospective observational study on adult patients hospitalized for COVID-19 is to assess for longitudinal changes in the upper respiratory microbiome during hospitalization and COVID-19 treatment. The secondary objectives of the study are to determine association between upper respiratory tract microbiome and severity of COVID-19 as well as its potential confounders and to compare SARS-CoV-2 viral load and plasma cytokine with upper respiratory tract microbiota in COVID-19.

## 2 Methods

### 2.1 Study design and subject recruitment

This was a prospective observational study on adult (age ≥ 18 years old) hospitalized patients who tested positive for SARS-CoV-2 on reverse transcription polymerase chain reaction (RT-PCR). Patients were included if they had at least one nasopharyngeal swab and throat swab (NPSTS) sample taken during hospitalization within 3 weeks of hospital admission after informed consent. Patients who were previously vaccinated against SARS-CoV-2, received antibiotics 3 months prior to hospitalization, or had missing data on clinical severity or antimicrobial therapy were excluded. Samples that were inadequate for DNA extraction and 16S rRNA gene amplicon sequencing for microbiota profiling were also excluded. Blood samples for cytokine profiling were taken as early as possible after hospital admission. Mechanically ventilated intensive care unit (ICU) patients without COVID-19 and healthy volunteers working in the same hospital environment were recruited for controls. This study was approved by The Joint Chinese University of Hong Kong – New Territories East Cluster Clinical Research Ethics Committee (2020.076).

### 2.2 Severity of COVID-19

COVID-19 severity was classified as asymptomatic, mild/moderate, or severe/critical based on the highest severity level at hospital discharge as previously described (28). Medical records including clinical notes, imaging, laboratory results, and observation charts were manually reviewed to determine the severity of COVID-19 according to the following criteria: Asymptomatic patients had no symptoms despite SARS-CoV-2 infection. Mild/moderate group included patients who had symptoms of fever, cough, myalgia, sore throat, and rigors related to SARS-CoV-2 infection but did not require oxygen therapy. Severe/critically ill patients with COVID-19 included those who had dyspnea, respiratory rate ≥ 30, or required oxygen therapy or mechanical ventilation for SARS-CoV-2 infection due to respiratory failure.

### 2.3 Respiratory sampling

Serial NPSTSs were collected for SARS-CoV-2 viral load quantification and 16S rRNA sequencing analysis. During the early COVID-19 pandemic, all patients with confirmed COVID-19 were hospitalized as part of the Hong Kong public health strategy. Patients were only discharged after testing negative for SARS-CoV-2 on RT-PCR. Patients who were asymptomatic or had mild disease were, sometimes, hospitalized for longer than 2 weeks despite clinical recovery. Therefore, serial NPSTS samples could be collected from all severity groups during hospitalization. The time points used in this study were the first sample within the first week of hospitalization and the second sample between the second and third weeks of hospitalization. Samples were stored in −80°C for 0.1–2.5 years until completion of recruitment for further analysis.

### 2.4 SARS-CoV-2 viral load quantification

Total RNA was extracted from mixed NPSTSs using the QIAamp Viral RNA Mini Kit (QIAGEN, Hilden, Germany). Primer–probe set targeting the N gene (2019-nCoV_N1-F: 5′-GAC CCC AAA ATC AGC GAA AT-3′; 2019-nCoV_N1-R: 5′-TCT GGT TAC TGC CAG TTG AAT CTG-3′; and 2019-nCoV_N1-P: 5′-FAM-ACC CCG CAT TAC GTT TGG TGG ACC-BHQ1-3′) was used to detect SARS-CoV-2 RNA by real-time RT-PCR as previously described (Lui et al., 2020). The detection limit of real-time RT-PCR was 694 copies/ml, and samples were considered negative if Ct values exceeded 39.9 cycles.

### 2.5 16S rRNA sequencing

Total DNA was extracted from mixed NPSTS using the QIAamp DNA Mini Kit (QIAGEN, Hilden, Germany) to characterize respiratory microbiota using 16S rRNA gene amplicon sequencing. The molecular process, including the DNA extraction, 16S PCR amplification, and library preparation, were performed at separate locations to avoid contamination. For quality control, negative controls (blank DNA extraction and PCR controls), positive controls (ZymoBIOMICS Microbial Community DNA Standard, catalog no. D6305), and technical replicates (randomly selected DNA samples) were also included. In brief, the 16S rRNA gene hypervariable V3-V4 region (~450 bp) was targeted (341F: 5′-CCT ACG GGN GGC WGC AG-3′; 806R: 5′-GGA CTA CNV GGG TWT CTA AT-3′), with barcodes indexed to each amplicon set for multiplexing sequencing on an Illumina MiSeq for PE300 reads (Chen et al., 2019). QIIME2 (v2022.2) with the latest SILVA ribosomal RNA database (v138 SSU Ref NR 99 dataset) was used to classify amplicon sequence variants (ASVs), with operational taxonomic table showing the proportion of bacterial reads per sample at different taxonomic levels after removing reads assigned to archaea, mitochondria, or chloroplasts.

### 2.6 Microbiota data analysis

Data distribution was assessed using Shapiro*–*Wilk test, and descriptive statistics such as mean and standard error as well as median and interquartile range (IQR) were used to summarize data. A phylogenetic tree was generated by inserting the representative reads into the SILVA 128 reference tree using the SATe-enabled phylogenetic placement method. Alpha diversity was assessed using Shannon, Simpson, richness, and evenness, and Wilcoxon rank sum test was used for assessing the pairwise difference between the defined groups. Beta diversity was assessed using unweighted and weighted GuniFrac and Bray–Curtis distance. Principal coordinate analysis was used to assess beta diversity between different groups using permutational multivariate analysis of variance (PERMANOVA) with 9,999 permutations using the *adonis2* in the Vegan R package (v2.6-4). In the effect size analysis using a single multivariable model, antibiotic-controlled association between metadata variables, including intubation, ICU, severity, peak CRP, hospitalized time, antivirus, peak viral load, Charlson comorbidity index, age, and gender, was tested by adding antibiotics into the model formula. An exploratory analysis on discriminative bacterial taxa between patients who required ICU admission and those that did not was estimated using linear discriminant analysis (LDA) effect size (LEfSe) with the default setting, with further comparisons of the relative abundances using nonparametric Mann–Whitney–Wilcoxon rank sum test and Tukey’s honest significant difference *post-hoc* test (Segata et al., 2011). Logistic regression and receiver operating characteristic (ROC) curve with the calculation of area under the ROC curve (AUC) were used to evaluate the potential markers identified for prediction of need for ICU admission. Delong’s test was used to assess the differences in AUCs. All other data visualization was performed using the ggplot package in R. A two-sided *p*-value < 0.05 or a false discovery rate–adjusted *p*-value (*p_adj_* or *q*) < 0.1 was used as the threshold for statistical significance.

### 2.7 Cytokine profile

A 3 ml of EDTA blood sample was taken from recruited patients with COVID-19 and immediately cooled and transported to the laboratory for processing. Plasma was separated by centrifugation (2,000g for 10 min) at 4°C and stored in 300 µl of aliquots at − 70°C until analysis. Milliplex human cytokine multiplex assay using the Bio-plex 200 System (Bio-Rad Laboratories, Inc. CA, USA) was used to determine levels of 32 cytokines including sCD40L, EGF, Eotaxin, FGF-2, Flt-3L, Fractalkine, GRO-α, IFN-α2, IFN-γ, IL-1β, IL-1RA, IL-3, IL-5, IL-6, IL-7, IL-8, IL-10, IL-12 p40, IL-12 p70, IL-13, IL-15, IL-18, IP-10, MCP-1, MCP-3, MDC, MIG, MIP-1β, TGF-α, TNF-α, TNF-β, and VEGF (Ling et al., 2021). Associations of cytokine factors with clinical variants and bacterial genera were analyzed by Fit a Negative Binomial Generalized Linear Model using the glm.nb in the MASS R package and a Spearman’s rank-order correlation test using the cor.test in the Stats R package, respectively.

## 3 Results

### 3.1 Cohort characteristics

A total of 314 NPSTS samples from 197 subjects that generated high-quality 16S sequence reads were analyzed (**Figure S1**). COVID-19 group consisted of 171 adult hospitalized patients (asymptomatic = 14, mild/moderate = 106, and severe/critical = 51) (**Table 1**). Meanwhile, 11 mechanically ventilated ICU adult hospitalized patients without COVID-19 and 15 adult healthy volunteers who worked in the same hospital as healthcare workers or departmental staff were included as controls. The baseline characteristics of the recruited subjects are shown in **Table 1** and **Table S1**. Overall, patients with severe/critical COVID-19 were older and were more likely to have comorbidities. Peak median viral load was similar across different COVID-19 severity (**Table 1**, *p* = 0.086). Antibiotic use was highest in patients with severe/critical COVID-19 and non–COVID-19 ICU patients. Use of specific COVID-19 treatments varied across the spectrum of COVID-19 severity.

**Table 1:** Cohort characteristics.

	Asymptomatic	Mild/Moderate	Severe/Critical	Non–COVID-19 ICU	Healthy Controls	p
	(N = 14)	(N = 106)	(N = 51)	(N = 11)	(N = 15)	
**Median Age, years**	32 (25–40)	49.0 (33–61)	66 (57–73)	62 (49–69)	42 (35–48)	<0.001
**Female Gender (%)**	6 (42.9)	60 (56.6)	16 (31.4)	3 (27.3)	10 (66.7)	0.012
Charlson Comorbidity Index						
None	11 (78.6)	49 (46.2)	3 (5.9)	2 (18.2)	12 (80.0)	<0.001
Mild (1–2)	3 (21.4)	42 (39.6)	20 (39.2)	5 (45.5)	3 (20.0)	0.397
Moderate (3–4)	0 (0)	10 (9.4)	21 (39.2)	4 (36.4)	0 (0.0)	<0.001
Severe (≥5)	0 (0)	5 (4.7)	8 (15.7)	0 (0.0)	0 (0.0)	0.036
Comorbidity (%)						
Good past health	11 (78.6)	65 (61.3)	14 (27.5)	4 (36.4)	14 (93.3)	<0.001
Cardiovascular diseases	2 (14.3)	31 (29.2)	32 (62.8)	6 (54.5)	0 (0)	<0.001
Chronic kidney diseases	0 (0)	3 (2.8)	3 (5.9)	0 (0)	0 (0)	0. 616
Chronic lung diseases	1 (7.1)	3 (2.8)	2 (3.9)	0 (0)	0 (0)	0.779
Diabetes mellitus	0 (0)	15 (14.2)	20 (39.2)	3 (27.3)	1 (6.7)	<0.001
Immunodeficiency	0 (0)	1 (0.9)	0 (0)	0 (0)	0 (0)	0.93
Liver diseases	1 (7.1)	6 (5.7)	5 (9.8)	1 (9.1)	0 (0)	0.703
Malignancy	0 (0)	2 (1.9)	4 (7.8)	0 (0)	0 (0)	0.222
**Bacterial Coinfection (%)**	0 (0)	1 (0.9)	9 (17.6)	1 (9.1)	–	0.0149
**Median Peak Viral Load (Ct)**	27.3 (20.3–29.6)	21.5 (17.1–27.6)	20.2 (18.2–25.5)	–	–	0.086
Treatment (%) ^b^						
Antibiotics	1 (7.1)	17 (16.0)	38 (74.5)	10 (90.9)	0 (0)	<0.001
Dexamethasone	0 (0.0)	7 (6.6)	47 (92.2)	0 (0)	0 (0)	<0.001
Remdesivir	0 (0.0)	9 (8.5)	38 (74.5)	0 (0)	0 (0)	<0.001
Lopinavir/ritonavir	0 (0.0)	28 (26.4)	14 (27.5)	0 (0)	0 (0)	0.009
Tocilizumab	0 (0.0)	2 (1.9)	2 (3.9)	0 (0)	0 (0)	0.78
Interferon beta-1b	0 (0.0)	39 (36.8)	23 (45.1)	0 (0)	0 (0)	<0.001
Outcomes (%)						
ICU admission	0 (0.0)	0 (0.0)	32 (62.7)	11 (100)	–	<0.001
Vasopressors	0 (0.0)	0 (0.0)	20 (39.2)	4 (36.4)	0 (0)	<0.001
Mechanical ventilation	0 (0.0)	0 (0.0)	20 (39.2)	11 (100)	0 (0)	<0.001
Hospital mortality	0 (0.0)	0 (0.0)	6 (11.8)	1 (9.1)	0 (0)	0.001

Bacterial coinfection was defined as positive growth of a bacterial pathogen in respiratory tract or blood culture samples within 48 hours of hospitalization. Values are expressed as median and (interquartile range) unless otherwise specified.

### 3.2 Upper airway microbiota in earliest samples of all subjects

**Figure 1:**
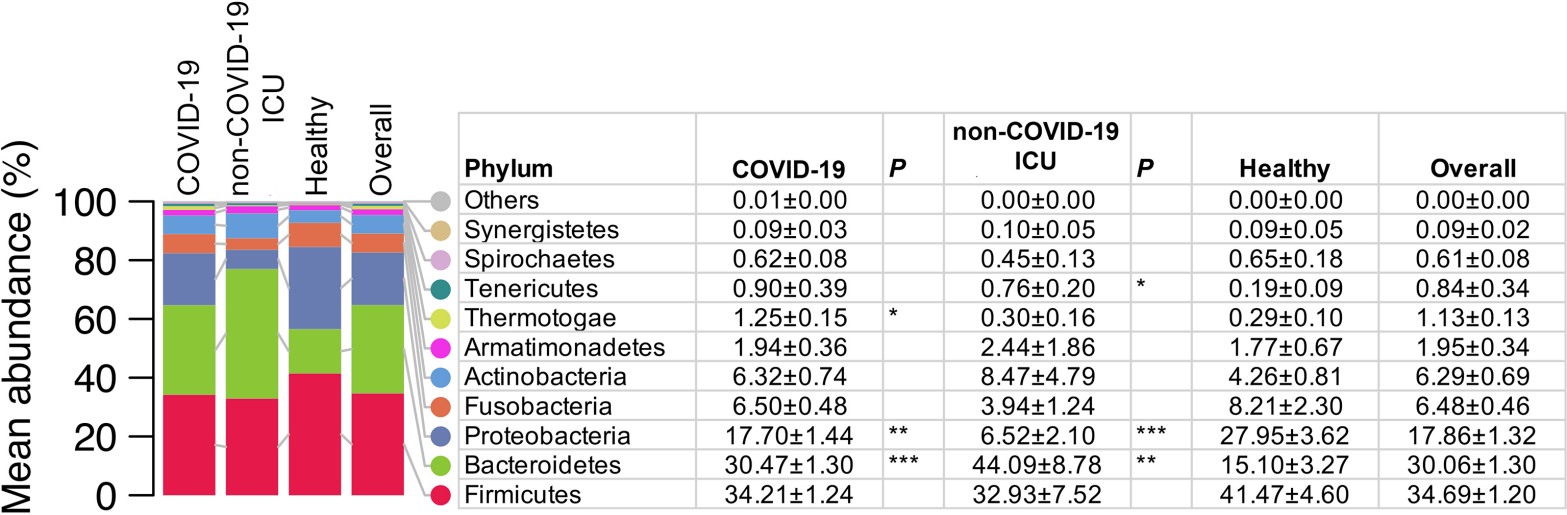
Comparison of the upper airway microbiota summarized at the phylum level from COVID-19 (n = 171), non–COVID-19 ICU patients (n = 11), and healthy controls (n = 15). Values in the table are mean abundance ± standard error of the mean. Phyla with a mean total relative abundance < 0.1% are grouped as others (Deinococcus_Thermus, Chlamydiae, Planctomycetes, and Chloroflexi). Wilcoxon rank sum (MWU) tests for the difference in relative abundance between hospitalized patients with COVID and healthy controls, and between non–COVID-19 ICU patients and healthy controls were performed. *p < 0.05, **p < 0.01, and ***p < 0.001.

We selected the earliest collected NPSTS samples from each subject (hospitalized COVID-19 = 171, non–COVID-19 ICU = 11, and healthy = 15) to profile the upper airway microbial communities. The median time interval between time of hospitalization and sampling time of first sample was 3 days. A total of 2,649,726 high-quality 16S rRNA V3-V4 reads were generated, ranging between 1,026 and 118,093 reads per sample (13,450 ± 11,799). As shown in **Figure 1** and **Figure S2**, *Firmicutes* was the most abundant microbial phyla in the surveyed samples (mean relative abundance ± SD of 34.70 ± 1.20%), followed by *Bacteroidota* (30.06 ± 1.30%), *Proteobacteria* (17.86 ± 1.32%), and 11 other phyla. *Proteobacteria* were lower in both hospitalized COVID-19 (17.70 ± 1.44%, Mann–Whitney U-test, *p* < 0.01) and non–COVID-19 ICU (6.52 ± 2.10%, *p <*0.001) patients compared with healthy volunteers (27.95 ± 3.62%). In contrast, *Bacteroidetes* was higher in both in both hospitalized COVID-19 (30.47 ± 1.30%, *p* < 0.001) and non–COVID-19 ICU (44.09 ± 8.78%, *p* < 0.01) patients compared with healthy volunteers (30.06 ± 1.30%).

**Figure 2:**
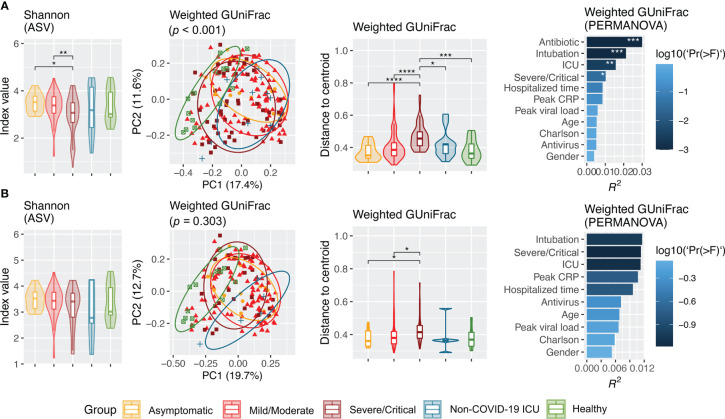
Alpha (Shannon) and beta (weighted GUniFrac) diversity of upper airway microbiota across cohort groups from all samples **(A)** and inclusion of only samples taken prior to antimicrobial therapy **(B)**. Panel **(A)** included samples from hospitalized patients with COVID-19 (n = 171, including 14 asymptomatic, 106 mild/moderate, and 51 severe/critical), non–COVID-19 patients (n = 11), and healthy controls (n = 15). Panel **(B)** shows difference in alpha and beta diversity when samples taken after antimicrobial therapy were excluded, which consisted of hospitalized COVID patients (n = 137, including 14 asymptomatic, 98 mild/moderate, and 25 severe/critical patients), non–COVID-19 ICU patients (n = 5), and healthy controls (n = 15). Difference in Shannon index was assessed by pairwise differences between groups using Wilcoxon rank sum test. Principal coordinate analysis was based on weighted GUniFrac inferred from amplicon sequence variants. Difference in weighted GUniFrac among groups was evaluated using permutational multivariate analysis of variance (PERMANOVA) with 9,999 permutations. Effect size (R^2^ value) of covariates on upper airway microbiota structure in patients with COVID-19 in the multivariable model. Antibiotic-controlled association between metadata variables (intubation, ICU, severity, peak CRP, hospitalized time, antivirus, peak viral load, Charlson comorbidity index, age, and gender) was tested by adding antibiotics into the multivariable model formula. *p < 0.05, **p < 0.01, ***p < 0.001, and ****p < 0.0001.

Alpha diversity at the ASV level as measured by Shannon, Simpson, and evenness was progressively reduced as severity of COVID-19 increased (**Figure 2A**, **Figure S3A**). Similarly, distinct clustering of the microbial communities from patients with COVID-19, particularly those with severe/critical severity, from healthy controls was observed in weighted GUniFrac, unweighted GUniFrac, and Bray–Curtis distance at the ASV level (*p* < 0.001). Antibiotic treatment (yes vs. no) was consistently the most significant covariate that had the largest effect on the overall structure of the upper airway microbiota in all beta diversity metrics (unweighted GUniFrac: *R^2 =^* 0.0442, *p* < 0.001; weighted GUniFrac: *R^2 =^* 0.0307, *p* < 0.001; and Bray–Curtis: *R^2 =^* 0.0216, *p* < 0.001) (**Figure 2A**, **Figure S3B**, **Table S2**). Similarly, the microbial community was also significantly affected by intubation (yes vs. no) and ICU admission (yes vs. no). The effect of severity (severe/critical vs. non-severe/critical), peak CRP (≤ 7 vs. > 7), hospitalized time (≤ 1 vs. ≥ 2 weeks), and antivirus treatment (yes vs. no) on structure of microbial community was not detected in all measures of beta diversity (**Figure 2A**, **Figure S3C**). Notably, 86.7% (OR = 40.3; 95% CI, 8.3–392.5; *p* < 0.001) and 62.5% (OR = 14.5; 95% CI, 5.5–40.6; *p* < 0.001) of hospitalized patients with COVID-19 who were intubated and admitted to the ICU, respectively, were treated with antibiotics prior to NPSTS sample collection in this study.

### 3.3 Upper airway microbiota in samples without antimicrobial use

Because antimicrobial use was the most significant factor that affected upper airway microbiota dysbiosis, we further assessed the difference in microbiota without the effect of antimicrobials. Removal of NPSTS samples taken after antimicrobial use resulted in 157 samples from 137 antibiotic-naïve hospitalized patients with COVID-19 (14 asymptomatic, 98 mild/moderate, and 25 severe/critical cases), five non–COVID-19 ICU patients, and 15 healthy volunteers. The median time interval between time of hospitalization and sampling time of first sample was 3 days. All measures of alpha diversity were no longer significantly different across COVID-19 severity groups when confounding by antimicrobial was removed (**Figure 2B**, **Figure S4A**). After exclusion of the samples taken after antimicrobials, microbiota from patients with severe/critical COVID-19 still showed dispersive distribution by beta diversity analysis when compared with other groups, but the differences were reduced (**Figure 2B**, **Figure S4B**). After removal of the samples taken after antimicrobial use, the confounding effect of intubation and ICU admission on upper respiratory microbiota in COVID-19 was no longer consistently found (**Figure S4C**).

### 3.4 Longitudinal changes in microbiota during hospitalization

**Figure 3:**
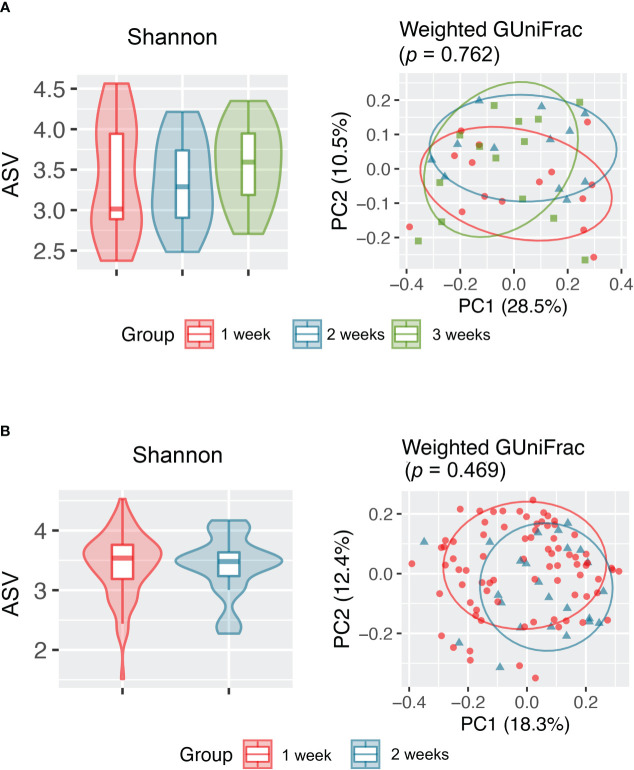
Alpha (Shannon index) and beta (weighted GUniFrac) diversity analyses revealed no significant difference in the upper respiratory tract microbiota between samples collected at different time points from **(A)** healthy individuals and **(B)** antibiotic-naïve patients with COVID-19.

Because upper airway microbiota is significantly confounded by use of antimicrobial use, longitudinal change in microbiota during hospitalization was assessed after exclusion of samples taken after antimicrobial therapy (102 samples). Assessment of these samples showed that upper airway microbiota alpha and beta diversity did not change overtime during 2 weeks of hospitalization in antibiotic-naïve hospitalized patients with COVID-19 and healthy individuals (**Figure 3**, **Figure S5**).

### 3.5 Association of upper airway microbiota dysbiosis with clinical features and ICU admission of patients with COVID-19

**Figure 4:**
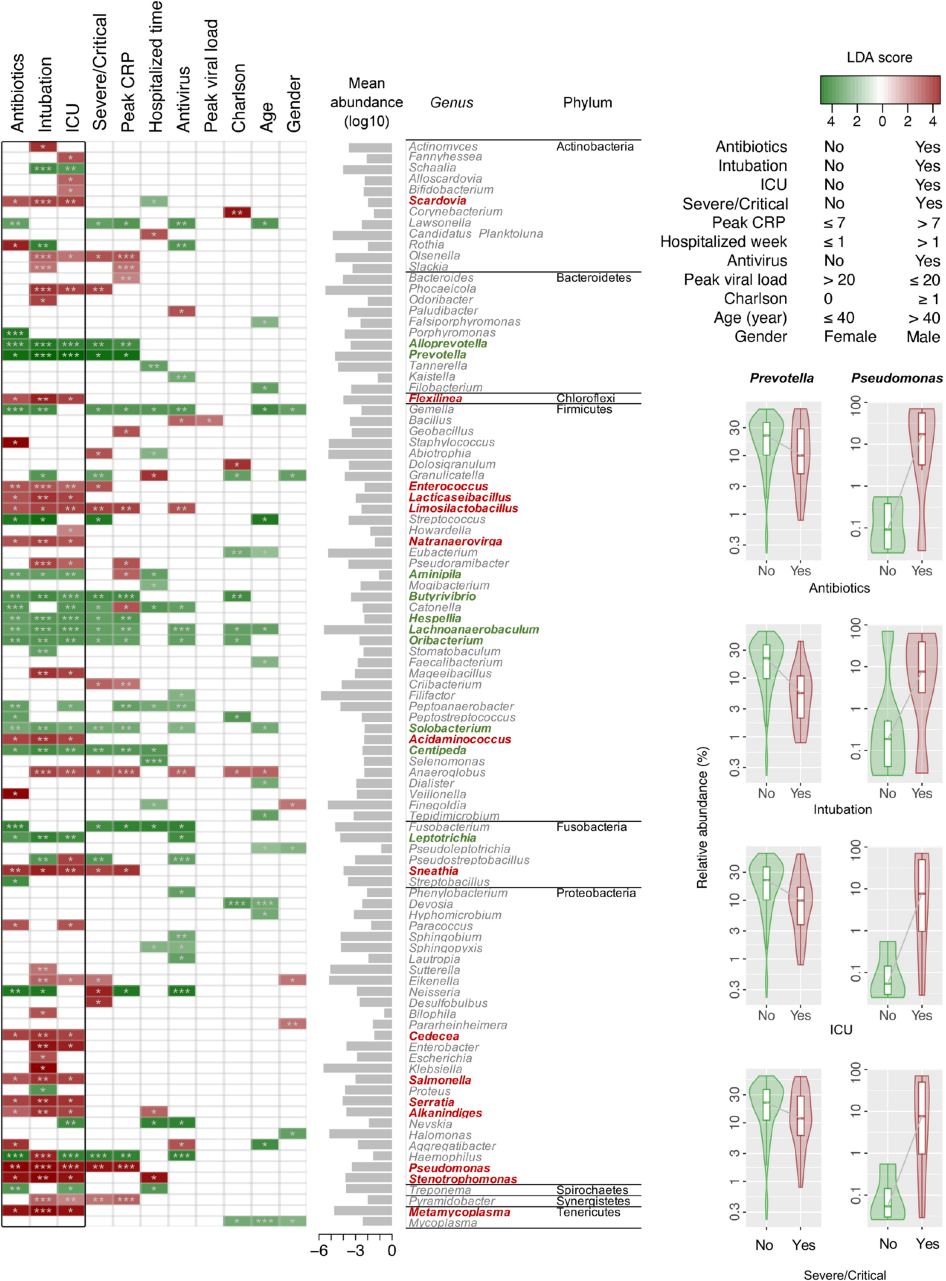
Linear discriminant analysis (LDA) effect size (LEfSe) identified discriminative bacterial genera associated with clinical variants in hospitalized patients with COVID-19 (n = 171). Names in red and green indicate bacterial genera with increased and decreased abundance, respectively, that were commonly associated with antibiotics, intubation, and ICU admission. Differences in the relative abundances of two representative bacterial genera (*Prevotella* and *Pseudomonas*) associated with antibiotics, intubation, ICU admission, and severe/critical COVID-19 are shown in the right panel of the figure. *p < 0.05, **p < 0.01,and ***p < 0.001.

**Figure 5:**
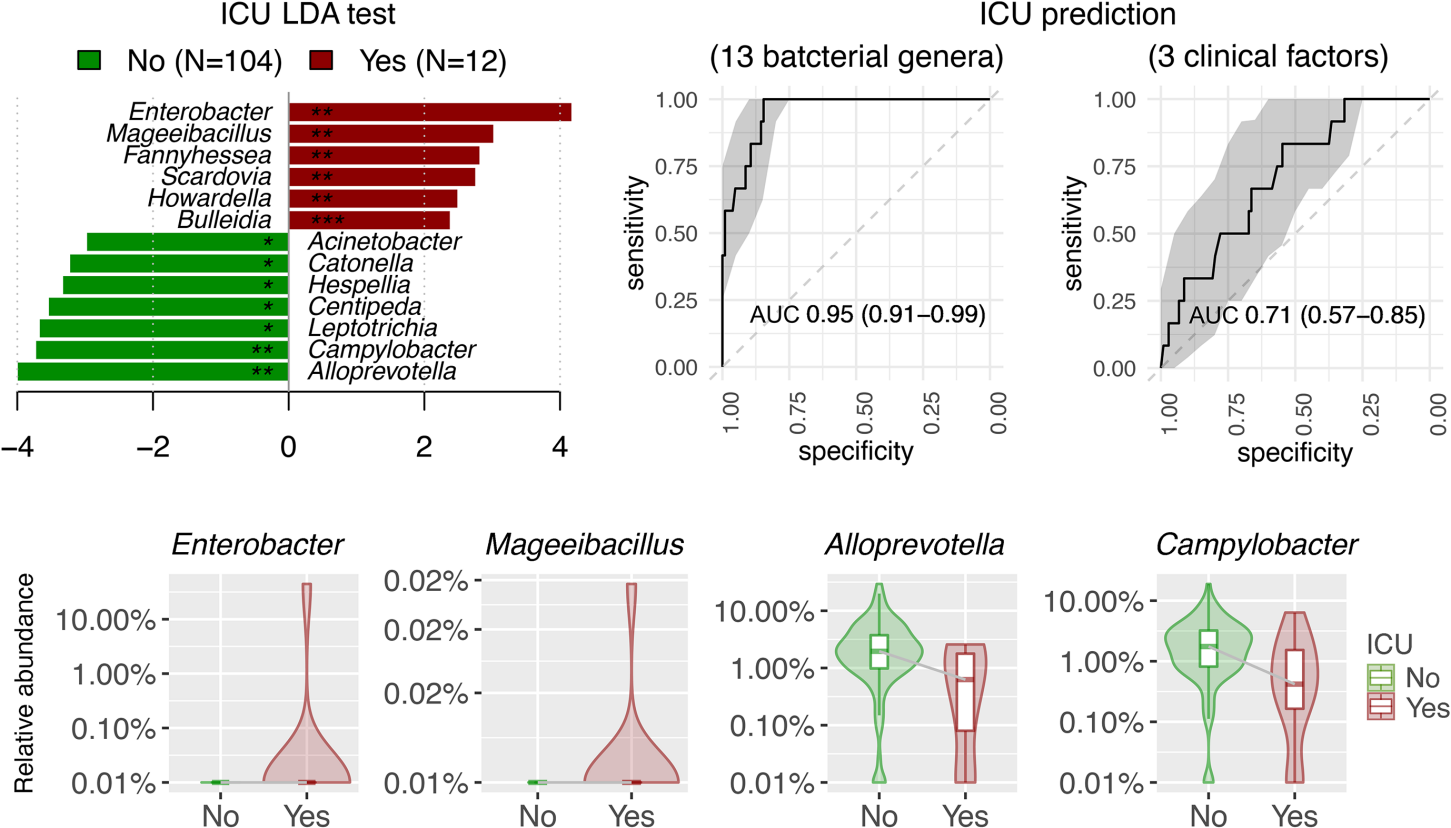
Linear discriminant analysis (LDA) effect size (LEfSe) showed the potential of 13 discriminative bacterial genera taken within the first week of hospitalization as predictor for ICU admission in antibiotic-naïve patients with COVID-19 (n = 116). Three clinical factors for ICU prediction included Charlson comorbidity index, age, and gender. AUC were expressed as AUC (95%CI).

Differentially abundant bacterial genera associated with demographic and clinical variants were characterized using LDA by LEfSe in the surveyed NPSTS samples from patients with COVID-19 in an exploratory analysis (n = 171) (LDA score > 2, *p* < 0.05) (**Figure 4**, **Table S3**). Interestingly, 15 and 10 bacterial genera showed consistent increase or decrease in the relative abundance in patients with antimicrobial use, intubation, and/or ICU admission. Among these, the enrichment of four bacterial genera (*Enterococcus*, *Limosilactobacillus*, *Sneathia*, and *Pseudomonas*) and the depression of eight bacterial genera (*Alloprevotella*, *Prevotella*, *Butyrivibrio*, *Hespellia*, *Lachnoanaerobaculum*, *Oribacterium*, *Solobacterium*, and *Centipeda*) were also significantly associated with the severity of patients with COVID-19. Because 63% (32 of 51) of patients with severe/critical COVID-19 in this study were admitted to the ICU, we further selected early NPSTS samples prior to antimicrobial use within the first week of hospitalization (n = 116) to identify bacterial markers that may be used to predict need for ICU admission (**Figure 5**). A total 13 discriminative bacterial genera were observed using LDA, with *Enterobacter*, *Mageeibacillus*, *Fannyhessea*, *Scardovia*, *Howardella*, and *Bulleidia* being higher but with *Alloprevotella*, *Campylobacter*, *Leptotrichia*, *Centipeda*, *Hespellia*, *Catonella*, and *Acinetobacter* being lower in patients who required ICU admission. The differential abundances of these 13 bacterial genera in the upper airway tract within the first week of hospitalization predicted the need for ICU admission with a combined AUC of 0.95 (95% CI of 0.91–0.99). Comparatively, prediction based on clinical demographics and comorbidity (age, gender, and Charlson comorbidity index) only achieved a combined AUC of 0.71 (95% CI of 0.57–0.85). Combination of clinical and microbiota (AUC of 0.96 (95% CI 0.93–1.00) did not improve on the predictive performance compared with the use of microbiota alone (**Figure S6**, *p* = 0.486).

### 3.6 Upper airway microbiota and plasma cytokine

**Figure 6:**
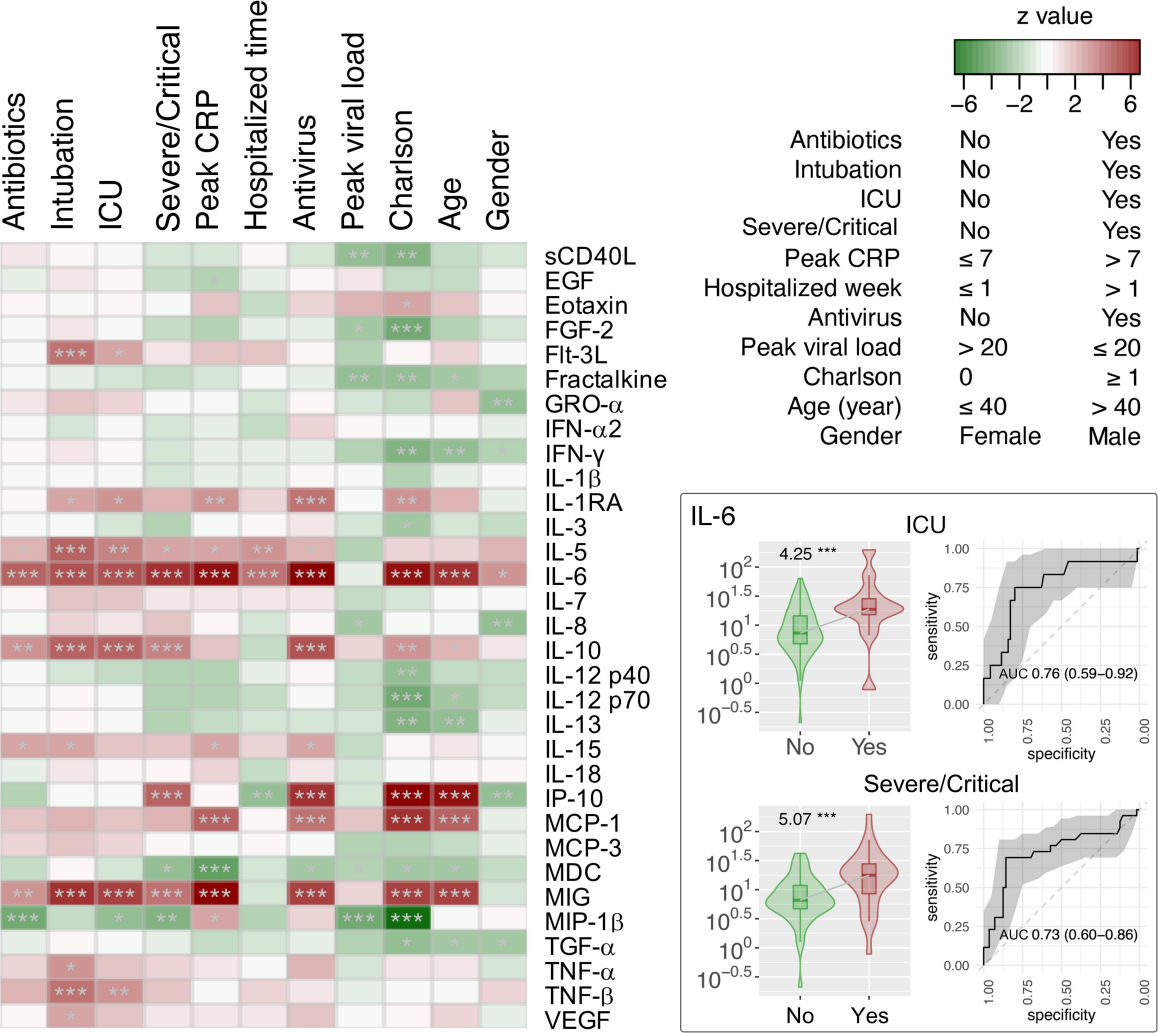
Association of cytokine factors with clinical variants in hospitalized patients with COVID-19 (n = 90). The z scores by the Fit a Negative Binomial Generalized Linear Model analysis using the glm.nb in the MASS R package were shown in the heat map. Relative abundance and receiver operating characteristic (ROC) analysis and area under the ROC curve (AUC) of IL-6 cytokine associated with ICU admission and severe/critical COVID-19 are shown in the right panel of the figure. *p < 0.05, **p < 0.01, and ***p < 0.001. AUC were expressed as AUC (95%CI).

**Figure 7:**
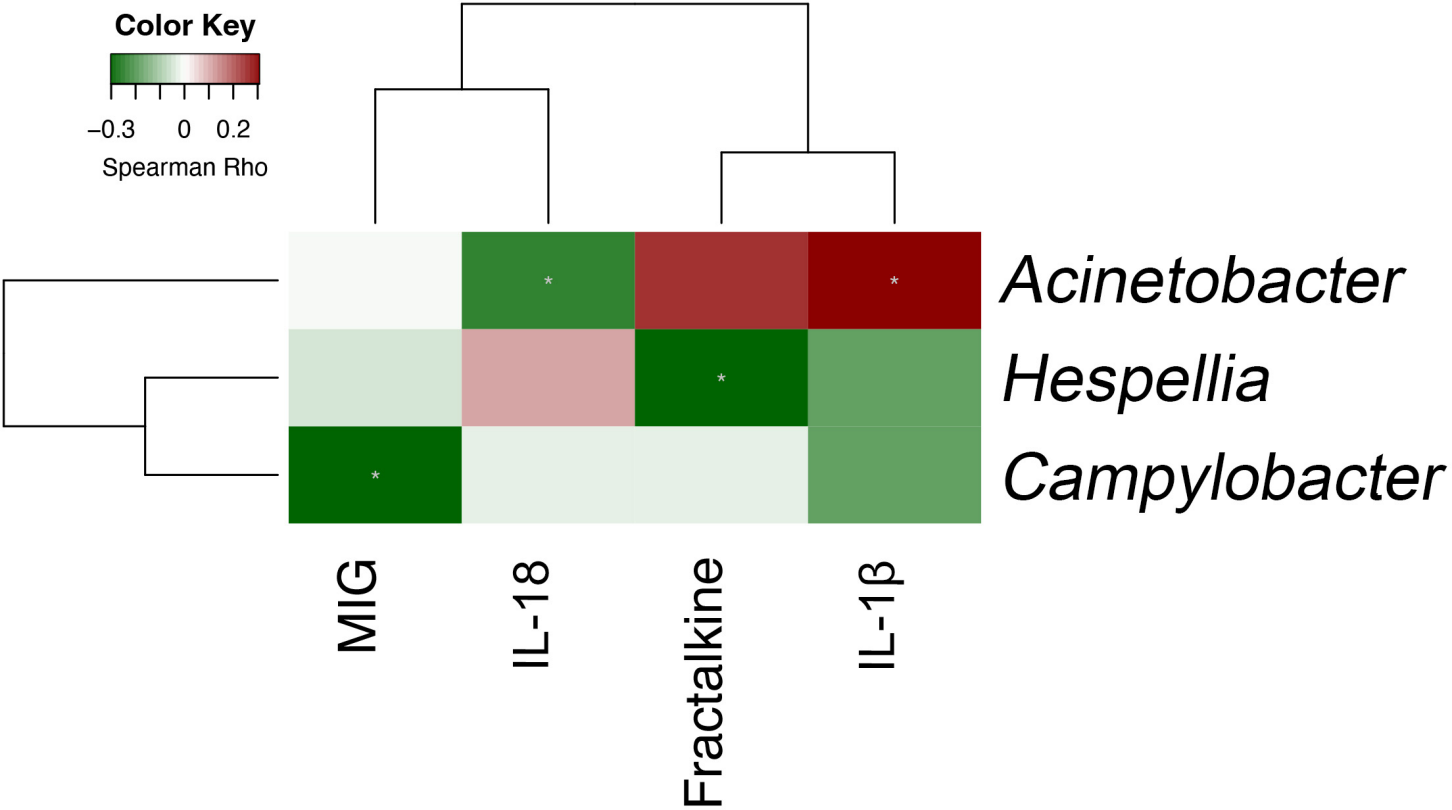
Spearman correlation between bacterial genera and plasma cytokines that are both individually associated with critical COVID-19 (n = 90).

Plasma was collected from 90 patients with COVID-19 to measure the levels of 32 cytokines. Median (IQR) time to cytokine profiling was 4 (2–8) days after hospital admission. Using the Negative Binomial Generalized Linear Model, changes in numerous cytokine factors were significantly associated with clinical factors (**Figure 6**). For example, increased IL-5, IL-6, IL-10, and MIG but deceased MIP-1β were observed in patients with severe/critical COVID-19 or those admitted to ICU, with satisfactory AUCs for predictive performance (**Table S4**). Those patients with higher IL-6 also had a higher chance of intubation and antibiotics and/or antivirus use. Older patients or those with higher Charlson comorbidity had higher levels of IL-6, IL-10, IP-10, MCP-1, and MIG, but lower levels of Fractalkine, IFN-χ, IL-12 p70, IL-13, and TGF-α. To understand the potential of upper airway microbiota dysbiosis on plasma cytokine levels, Spearman correlations between bacterial genera and cytokines were explored (**Figure S7**, **Table S5**). Next, we focused on the 13 bacterial genera that were associated with a need for ICU admission and found that only *Acinetobacter*, *Hespellia*, and *Campylobacter* were associated with MIG, IL-18, Fractalkine, and IL-1β after removal of samples taken after antimicrobial therapy (**Figure 7**).

## 4 Discussion

In this prospective, longitudinal observational study on upper airway microbiota in adult hospitalized patients with COVID-19, antimicrobial use accounted for most of the observed differences in microbiota during hospitalization and across severity groups. Alpha diversity in the upper airway was similar across severity of COVID-19 in the absence of antimicrobial use. In contrast, beta diversity was different between asymptomatic and severe/critical COVID-19 even in the absence of antimicrobial use. Upper airway microbiota in patients with COVID-19 remained unchanged during 2 weeks of hospitalization if antimicrobials were not used. Peak viral load was not associated with upper airway microbiota in COVID-19. Early hospitalization upper airway microbiota may be associated with severity of COVID-19.

Many studies have implicated that reduced alpha diversity in upper respiratory microbiome is a hallmark of higher severity of COVID-19 (Hernandez-Teran et al., 2021; Ma et al., 2021; Merenstein et al., 2021; Ren et al., 2021; Shilts et al., 2021; Ventero et al., 2021; Bradley et al., 2022; Chen et al., 2022; de Castilhos et al., 2022; Hurst et al., 2022; Merenstein et al., 2022). However, mechanical ventilation, duration of ICU admission, and antimicrobial use account for a substantial portion of the variations seen in upper respiratory tract microbiome in COVID-19 (Llorens-Rico et al., 2021; Ren et al., 2021; de Castilhos et al., 2022). Our study corroborates with these findings, as there was no difference alpha diversity between severe/critical and asymptomatic COVID-19 when confounding by use of antimicrobial was removed. These results are consistent with animal and human non–COVID-19 studies that generally demonstrated that antimicrobial use reduces bacterial alpha diversity (Huang et al., 2022; Kwon et al., 2022; Lekang et al., 2022). However, exclusion or statistical adjustment of samples for antimicrobial use was often not done or reported in COVID-19–related microbiome studies (Ma et al., 2021; Merenstein et al., 2021; Rueca et al., 2021; Ventero et al., 2021; Chen et al., 2022; Gauthier et al., 2022). As antimicrobial use is more common as COVID-19 severity increases, this may explain why many studies reported reduced alpha diversity as COVID-19 severity increased (Langford et al., 2021). Together, careful considerations on study design and data interpretation are required when assessing the validity of COVID-19–related microbiota study results.

Unlike alpha diversity that was mostly related to use of antimicrobials, we found that beta diversity was different across severity of COVID-19 even after removal of samples taken after antimicrobials. Overall, beta diversity of asymptomatic COVID-19 and severe/critical COVID-19 were closet and furthest from that of healthy individuals, respectively. In this study, *Enterococcus* in the upper airway was associated with COVID-19 severity and mechanical ventilation. Interestingly, this parallels the finding of increased relative abundance of *Enterococcus* in lungs of murine sepsis (Dickson et al., 2016b).

Nevertheless, association between specific bacterial genera and severity of COVID-19 has been inconsistently reported across different cohort studies (Merenstein et al., 2022; Candel et al., 2023). In our exploratory analysis, 13 bacterial genera from the upper airway microbiota were associated with a need for ICU admission. Although we were unable to perform internal validation, the lower abundance of upper airway *Alloprevotella* and *Campylobacter* in patients with COVID-19 requiring ICU admission found in this study was consistent other reports (Shilts et al., 2021; Smith et al., 2021; Chen et al., 2022; Hurst et al., 2022). Along the same lines, we found a lower abundance of *Acinetobacter* when COVID-19 severity was higher (Feehan et al., 2021; Smith et al., 2021). However, this has been inconsistently reported as some have found a higher abundance of *Acinetobacter* in upper airway microbiota in severe COVID-19 (Ma et al., 2021; Ren et al., 2021; Chen et al., 2022). Last, we showed that *Enterobacter* abundance was relatively higher in patients with COVID-19 admitted to ICU. Although this was also reported by Chen et al., not all studies supported the positive correlation between *Enterobacter* abundance and COVID-19 severity (Feehan et al., 2021; Chen et al., 2022; Gauthier et al., 2022). The reasons for these disparities are manifold. First, most of these studies did not exclude samples that were taken after antimicrobial therapy that may have confounded their findings. Second, tracheal intubation itself is associated with changes in upper and lower respiratory microbial diversity and may introduce bias in data interpretation (Kelly et al., 2016; Alagna et al., 2023). Third, there are baseline variations in microbiome composition and diversity among different ethnicity and geographic locations (Gupta et al., 2017).

Although some longitudinal studies reported changes in upper airway microbiota over time, many were confounded by medical interventions such as antimicrobial use and tracheal intubation (Llorens-Rico et al., 2021; Merenstein et al., 2021; Ren et al., 2021; Xu et al., 2021). In contrast, we showed that, in the absence of antimicrobial use, upper airway respiratory microbiome remained stable over 2 weeks of hospitalization in COVID-19 and healthy volunteers. Similarly, although some studies suggest viral load is associated with respiratory microbiome, we found that upper airway microbiota was unrelated to peak viral load. This maybe because we used serial samples to define peak viral load, whereas viral loads in other studies were determined by a single time point (Miller et al., 2021). It should be noted that severity of COVID-19 is more related to duration of viral shedding than peak viral load (Lui et al., 2020; Zheng et al., 2020).

The positive association between plasma cytokines such as IL-6, IL-10, IP-10, and MIG with COVD-19 severity is consistent with previous studies (Chi et al., 2020; Hadjadj et al., 2020; Zhao et al., 2020; Ling et al., 2021; Ochoa-Ramirez et al., 2022). In addition, specific correlations between upper respiratory tract microbiota and plasma cytokine levels were identified. Similar to previous reports, most of the pairwise associations were not directly between microbiota and cytokines that were individually associated with COVID-19 severity (Ren et al., 2021). This may have been due to confounding by antimicrobial therapy. Indeed, when effect of antimicrobial therapy was removed, the correlations were different between upper respiratory tract microbiota and plasma cytokine. Nevertheless, we found that only three of the 13 bacterial genera that may predict ICU admission were associated with plasma cytokines that were themselves associated with critical COVID-19. Furthermore, neither were the associations particularly strong. For example, we found that *Campylobacter* in the upper respiratory tract was inversely related to MIG and COVID-19 severity, but the relationship was relatively weak. Overall, the likely explanation is that plasma cytokine levels may not directly reflect the local inflammation profile in upper respiratory tract dysbiosis in COVID-19.

The main strength of this study is the comprehensive matching between all COVID-19 severity phenotypes, timing and type of medical intervention, and serial microbiota sampling. This enabled a robust analysis on relationship between microbiota and COVID-19 severity after exclusion of samples that may be affected by medical interventions. However, our study has several limitations. First, this was a single-center study on patients of Southeast Asian descent, which may limit the generalizability of our results. Second, we did not analyze viral or fungal microbiota. Third, we did not analyze lower respiratory tract samples that may be more closely related to severity of COVID-19. Fourth, like many COVID-19 microbiome studies, viral culture medium was used during sample collection rather than fresh sampling, which may have affected the results (Merenstein et al., 2022). Fifth, we did not analyze microbiota according to SARS-CoV-2 strain as variant typing was not performed for all cases. However, the omicron variant first circulated in Hong Kong only during first half-year of 2022. If variant type was defined by study recruitment date, then patients with omicron would represent less than 6% of the study cohort. Although the omicron variant is associated with less severe phenotype, our results are unlikely to be significantly confounded as most of the COVID-19 cases were of alpha and delta variants (Esper et al., 2022). Furthermore, all included cases were unvaccinated against SARS-CoV-2. Sixth, although this resulted in one of the largest studies on respiratory microbiota in COVID-19, the sample size was still relatively modest and may have limited the power to detect subtle differences in microbiota. Moreover, the exploratory findings on association between upper respiratory bacterial genera and severity of COVID-19 require future validation. Last, an absolute abundance was not assessed, and a subsequent compositional approach to assess respiratory microbiota in COVID-19 may be helpful (Gloor et al., 2017).

## 5 Conclusion

Upper respiratory microbiota in adult patients hospitalized for COVID-19 remains stable during the first two weeks of hospitalization in the absence of antimicrobial use. Beta diversity is different across spectrum of COVID-19 severity, whereas alpha diversity is similar. Early hospitalization upper airway microbiota may be associated with severity of COVID-19. Peak viral load was not associated with upper airway microbiota in COVID-19.

## Data availability statement

All sequence reads generated in this study have been deposited to the NCBI Sequence Read Archive (SRA) under Bioproject accession PRJNA934153.

## Ethics statement

The studies involving human participants were reviewed and approved by The Joint Chinese University of Hong Kong – New Territories East Cluster Clinical Research Ethics Committee. The patients/participants provided their written informed consent to participate in this study.

## Author contributions

LL, PC, CW, and ZC conceived and supervised the study. LL, W-TW, CL, and GL recruited study participants. SC, AC, LCC, LCSC, and JZ collected clinical information. AY and HC performed laboratory investigations. LL, PC, and ZC analyzed data and visualized results. LL and ZC wrote original draft. DH, CW, and PC reviewed and edited manuscript. All authors contributed to the article and approved the submitted version.

## Supplementary material

Supplementary Figure 1 Study recruitment flow chart showing inclusion and exclusion of study participants.

Supplementary Figure 2 Phylum abundance per subjective (n = 171) including hospitalized COVID-19 patients (14 asymptomatic, 106 mild/moderate, 51 severe/critical), 11 mechanically ventilated adult ICU patients without COVID-19 (non-COVID-19 ICU), and 15 adult healthy volunteers (Healthy).

Supplementary Figure 3 Upper airway microbiota dysbiosis associated with hospitalized COVID-19 patients (n = 171, including 14 asymptomatic, 106 mild/moderate and 51 severe/critical), non-COVID-19 patients (n = 11) and healthy controls (n = 15). **(A)** Comparison of the upper airway microbiota alpha diversity summarized at the amplicon sequence variant (ASV) level. Pairwise differences between groups were performed using Wilcoxon rank-sum test. **(B)** Principal coordinate analysis based on unweighted and weighted GUniFrac and Bray-Curtis distance metrics inferred from ASVs. Beta diversity among groups was evaluated using permutational multivariate analysis of variance (PERMANOVA) with 9,999 permutations. **(C)** Effect size (R^2^ value) of variables on the upper airway microbiota in the hospitalized COVID-19 patients. Antibiotic-controlled association between metadata variables (intubation, ICU, severity, peak CRP, hospitalized time, antivirus, peak viral load, Charlson’s comorbidity index, age and gender) were tested by adding antibiotics into the model formula. **p* < 0.05, ***p* < 0.01, ****p* < 0.001 and *****p* < 0.0001.

Supplementary Figure 4 Upper airway microbiota dysbiosis associated with antibiotic-naïve hospitalized COVID-19 patients (n = 137, including 14 asymptomatic, 98 mild/moderate, and 25 severe/critical patients), non-COVID-19 ICU patients (n = 5) and healthy controls (n = 15) at the time when samples were collected. **(A)** Comparison of the upper airway microbiota alpha diversity summarized at the amplicon sequence variant (ASV) level. Pairwise differences between groups were performed using Wilcoxon rank-sum test. **(B)** Principal coordinate analysis based on unweighted and weighted GUniFrac and Bray-Curtis distance metrics inferred from ASVs. Beta diversity among groups was evaluated using permutational multivariate analysis of variance (PERMANOVA) with 9,999 permutations. **(C)** Effect size (R^2^ value) of variables on the upper airway microbiota in the antibiotic-naïve hospitalized COVID-19 patients. *, *p* < 0.05; **, *p* < 0.01; ***, *p* < 0.001.

Supplementary Figure 5 Alpha and beta diversity analyses revealed no significant difference in the upper respiratory tract microbiota between samples collected at different time points from **(A)** healthy individuals and **(B)** antibiotic-naïve hospitalized COVID-19 patients.

Supplementary Figure 6 Predictive performance of 13 discriminative bacterial genera in upper respiratory tract microbiota and clinical factors (age, gender, Charlson’s comorbidity index) on need for ICU admission in hospitalized patients with COVID-19. Samples taken after antimicrobial therapy were excluded in this analysis. AUC were expressed as AUC (95%CI).

Supplementary Figure 7 Spearman correlation between bacterial genera and plasma cytokine in adult hospitalized COVID-19 patients (n = 90).
