# Supplementary figures and images for: Characterization of upper airway microbiome across severity of COVID-19 during hospitalization and treatment
Source: Front Cell Infect Microbiol. 2023 Jul 4;13:1205401. doi: 10.3389/fcimb.2023.1205401 (PMC10352853; doi:10.3389/fcimb.2023.1205401)

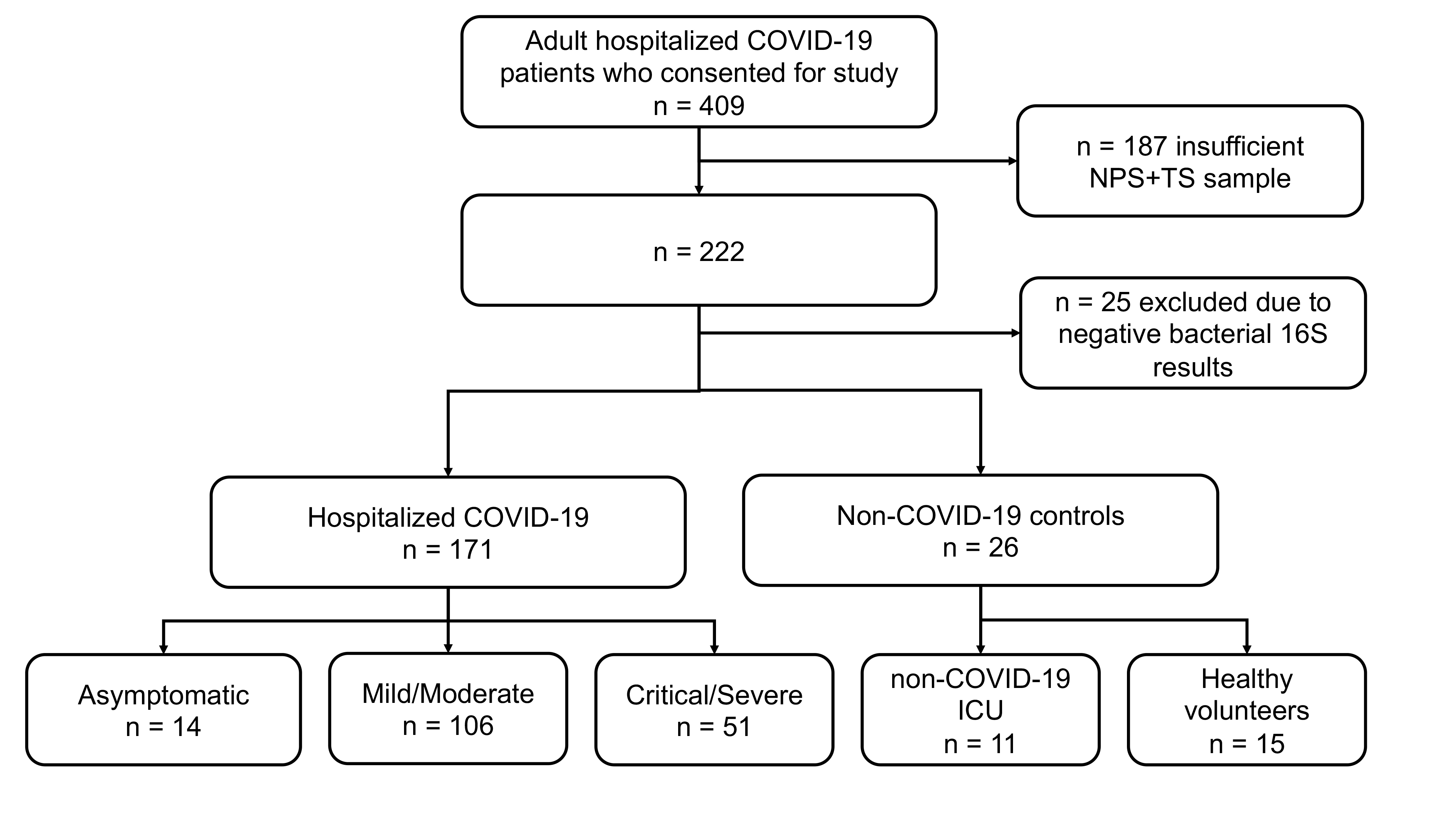

Supplement: Supplementary Figure 1 — Study recruitment flow chart showing inclusion and exclusion of study participants. [file Image_1.jpeg]

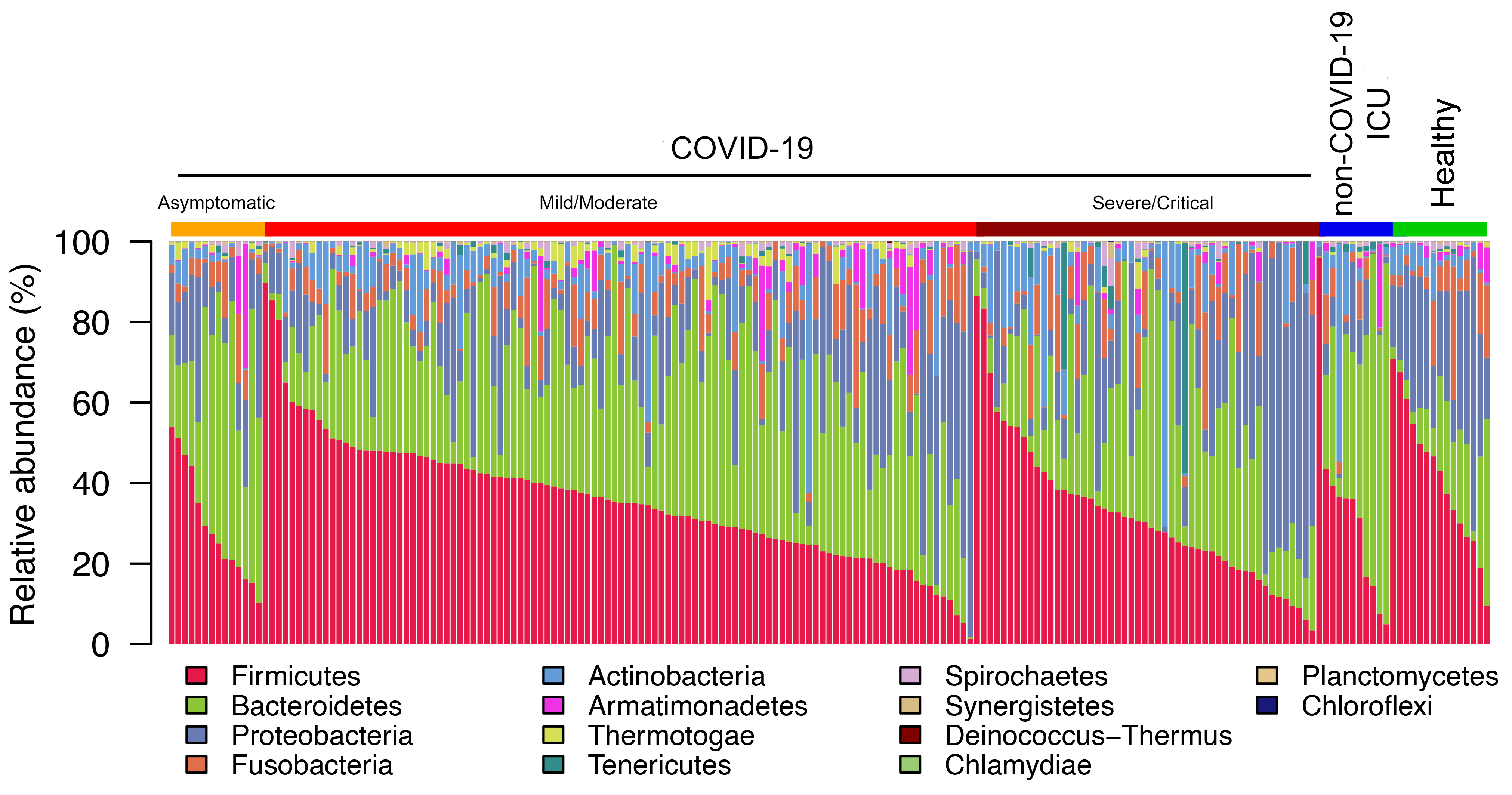

Supplement: Supplementary Figure 2 — Phylum abundance per subjective (n = 171) including hospitalized COVID-19 patients (14 asymptomatic, 106 mild/moderate, 51 severe/critical), 11 mechanically ventilated adult ICU patients without COVID-19 (non-COVID-19 ICU), and 15 adult healthy volunteers (Healthy). [file Image_2.jpeg]

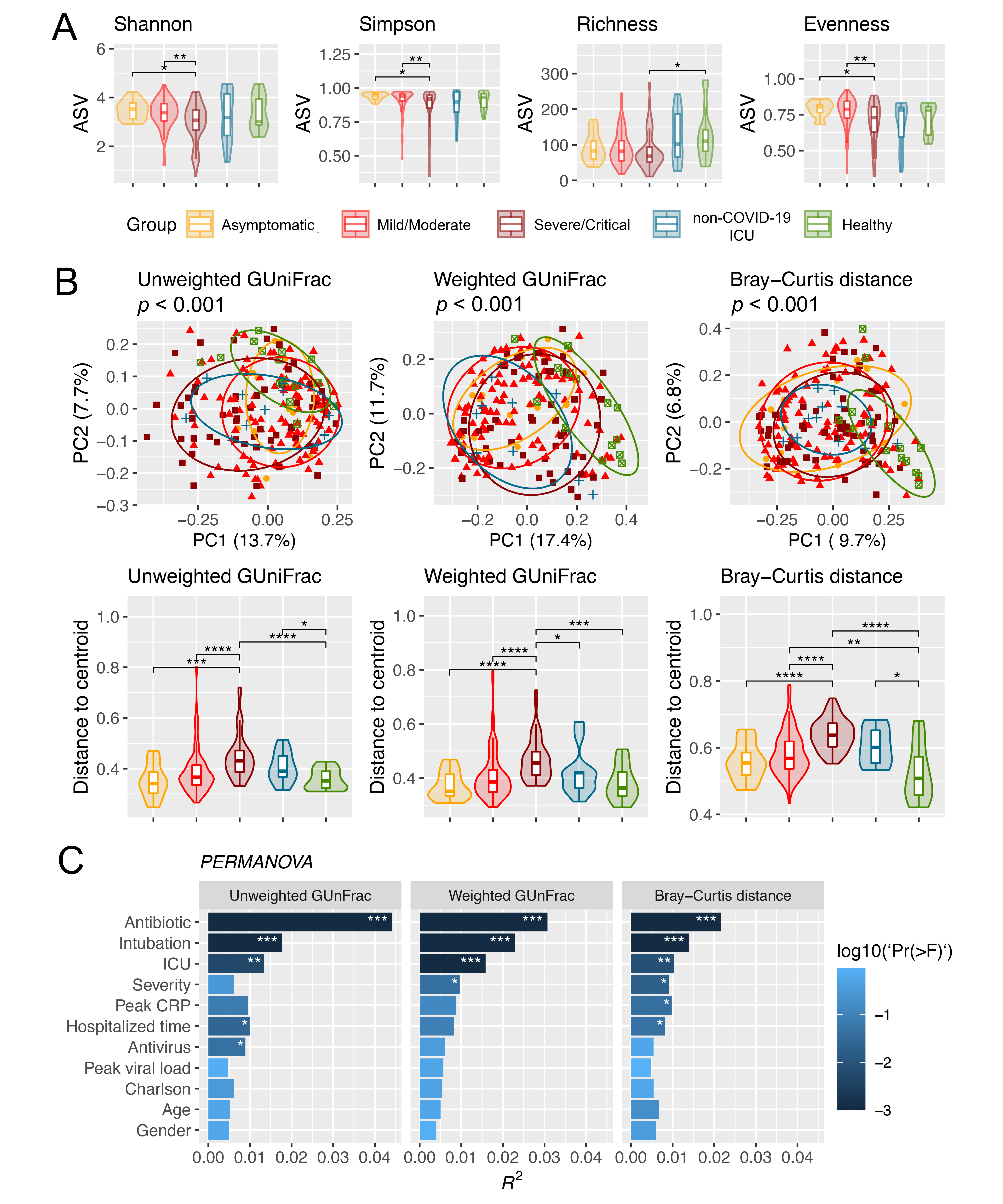

Supplement: Supplementary Figure 3 — Upper airway microbiota dysbiosis associated with hospitalized COVID-19 patients (n = 171, including 14 asymptomatic, 106 mild/moderate and 51 severe/critical), non-COVID-19 patients (n = 11) and healthy controls (n = 15). (A) Comparison of the upper airway microbiota alpha diversity summarized at the amplicon sequence variant (ASV) level. Pairwise differences between groups were performed using Wilcoxon rank-sum test. (B) Principal coordinate analysis based on unweighted and weighted GUniFrac and Bray-Curtis distance metrics inferred from ASVs. Beta diversity among groups was evaluated using permutational multivariate analysis of variance (PERMANOVA) with 9,999 permutations. (C) Effect size (R2 value) of variables on the upper airway microbiota in the hospitalized COVID-19 patients. Antibiotic-controlled association between metadata variables (intubation, ICU, severity, peak CRP, hospitalized time, antivirus, peak viral load, Charlson’s comorbidity index, age and gender) were tested by adding antibiotics into the model formula. *p < 0.05, **p < 0.01, ***p < 0.001 and ****p < 0.0001. [file Image_3.jpeg]

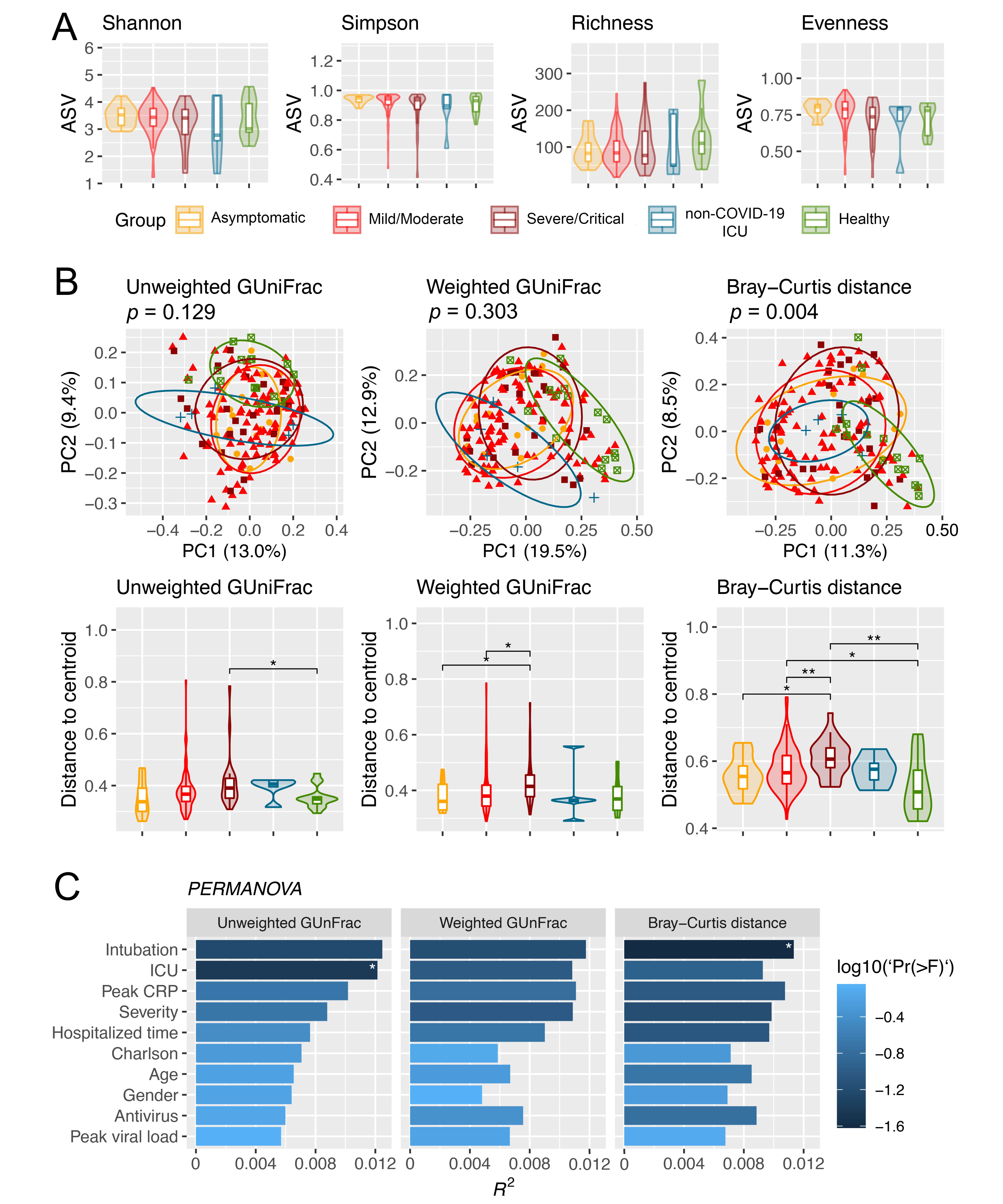

Supplement: Supplementary Figure 4 — Upper airway microbiota dysbiosis associated with antibiotic-naïve hospitalized COVID-19 patients (n = 137, including 14 asymptomatic, 98 mild/moderate, and 25 severe/critical patients), non-COVID-19 ICU patients (n = 5) and healthy controls (n = 15) at the time when samples were collected. (A) Comparison of the upper airway microbiota alpha diversity summarized at the amplicon sequence variant (ASV) level. Pairwise differences between groups were performed using Wilcoxon rank-sum test. (B) Principal coordinate analysis based on unweighted and weighted GUniFrac and Bray-Curtis distance metrics inferred from ASVs. Beta diversity among groups was evaluated using permutational multivariate analysis of variance (PERMANOVA) with 9,999 permutations. (C) Effect size (R2 value) of variables on the upper airway microbiota in the antibiotic-naïve hospitalized COVID-19 patients. *, p < 0.05; **, p < 0.01; ***, p < 0.001. [file Image_4.jpeg]

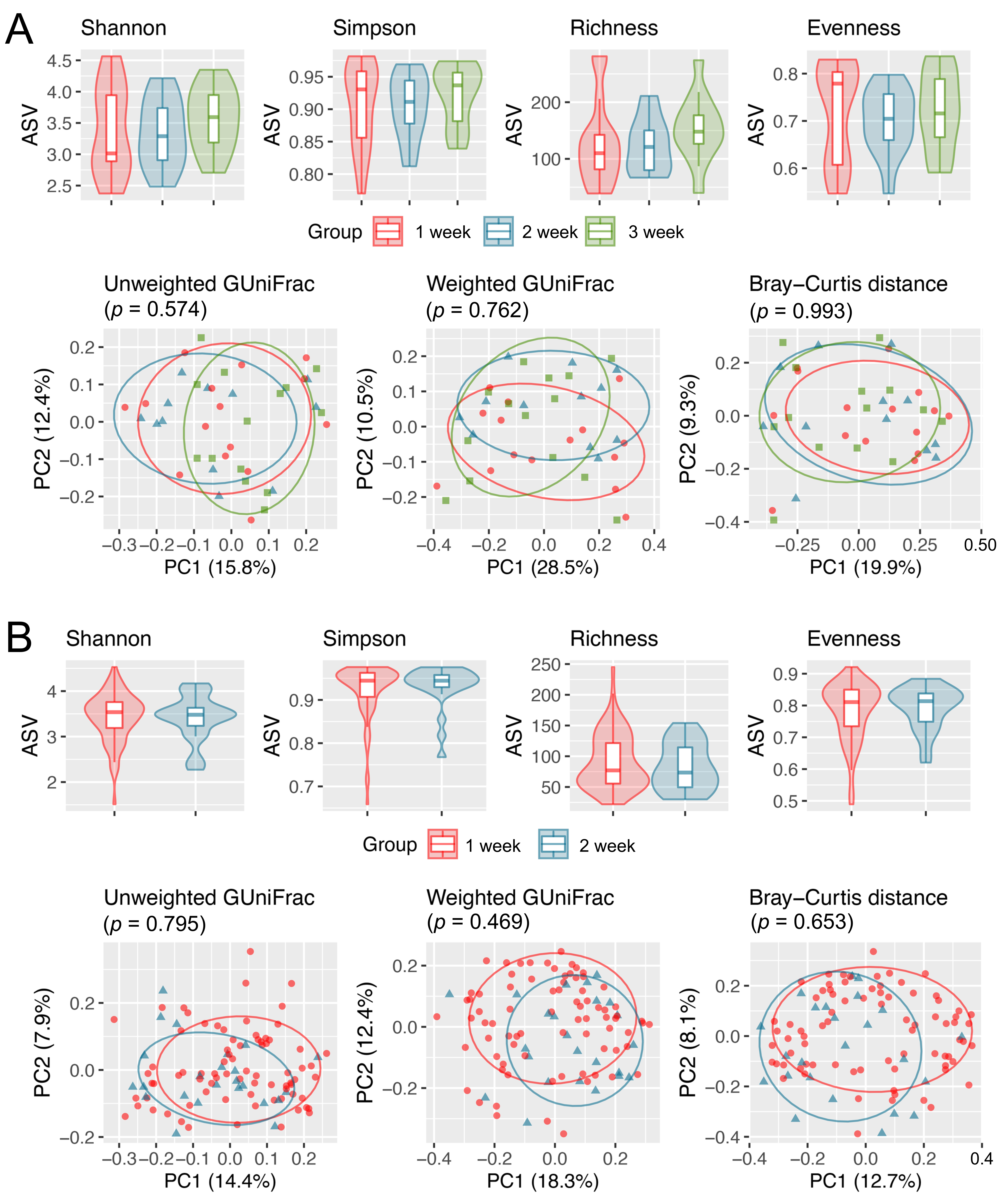

Supplement: Supplementary Figure 5 — Alpha and beta diversity analyses revealed no significant difference in the upper respiratory tract microbiota between samples collected at different time points from (A) healthy individuals and (B) antibiotic-naïve hospitalized COVID-19 patients. [file Image_5.jpeg]

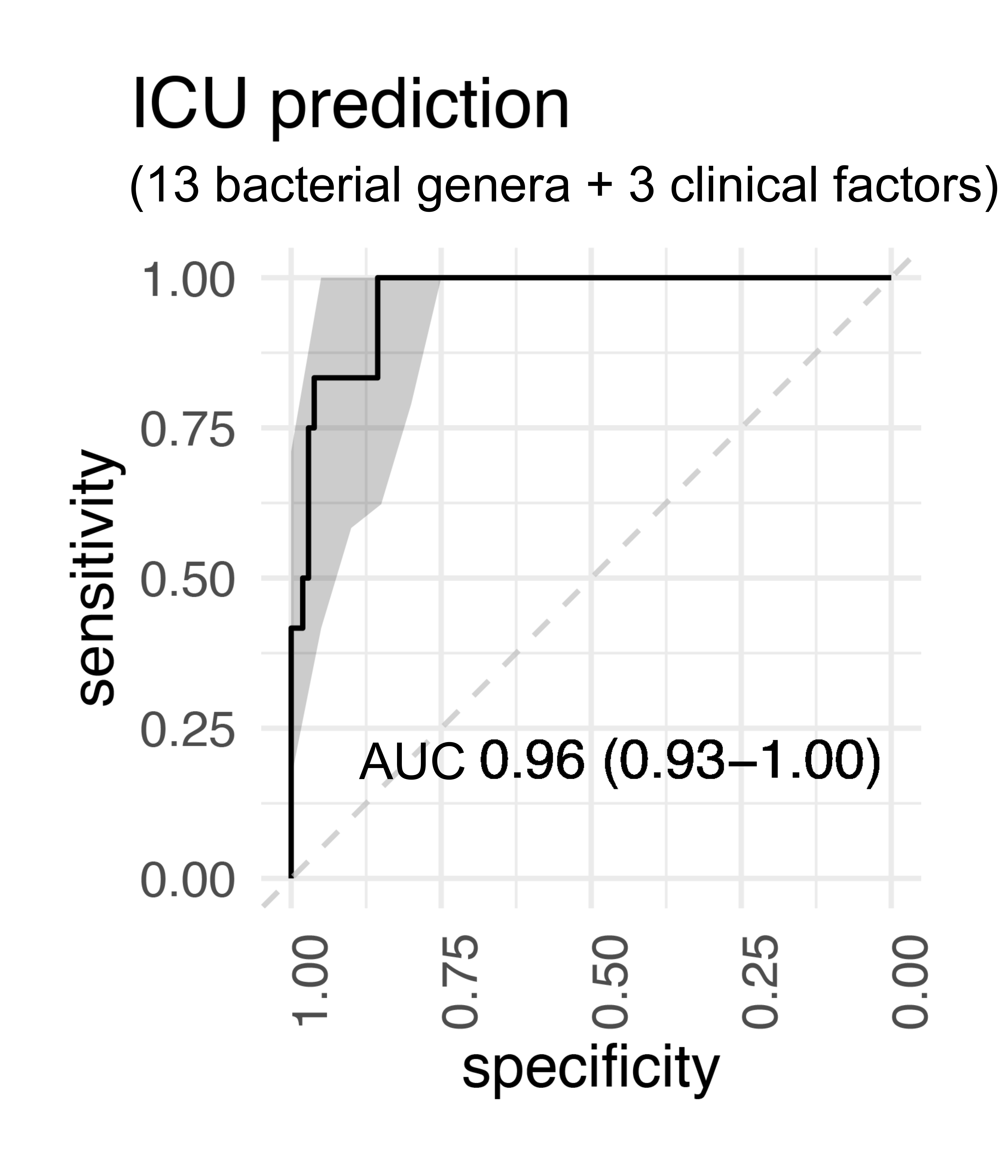

Supplement: Supplementary Figure 6 — Predictive performance of 13 discriminative bacterial genera in upper respiratory tract microbiota and clinical factors (age, gender, Charlson’s comorbidity index) on need for ICU admission in hospitalized patients with COVID-19. Samples taken after antimicrobial therapy were excluded in this analysis. AUC were expressed as AUC (95%CI). [file Image_6.jpeg]

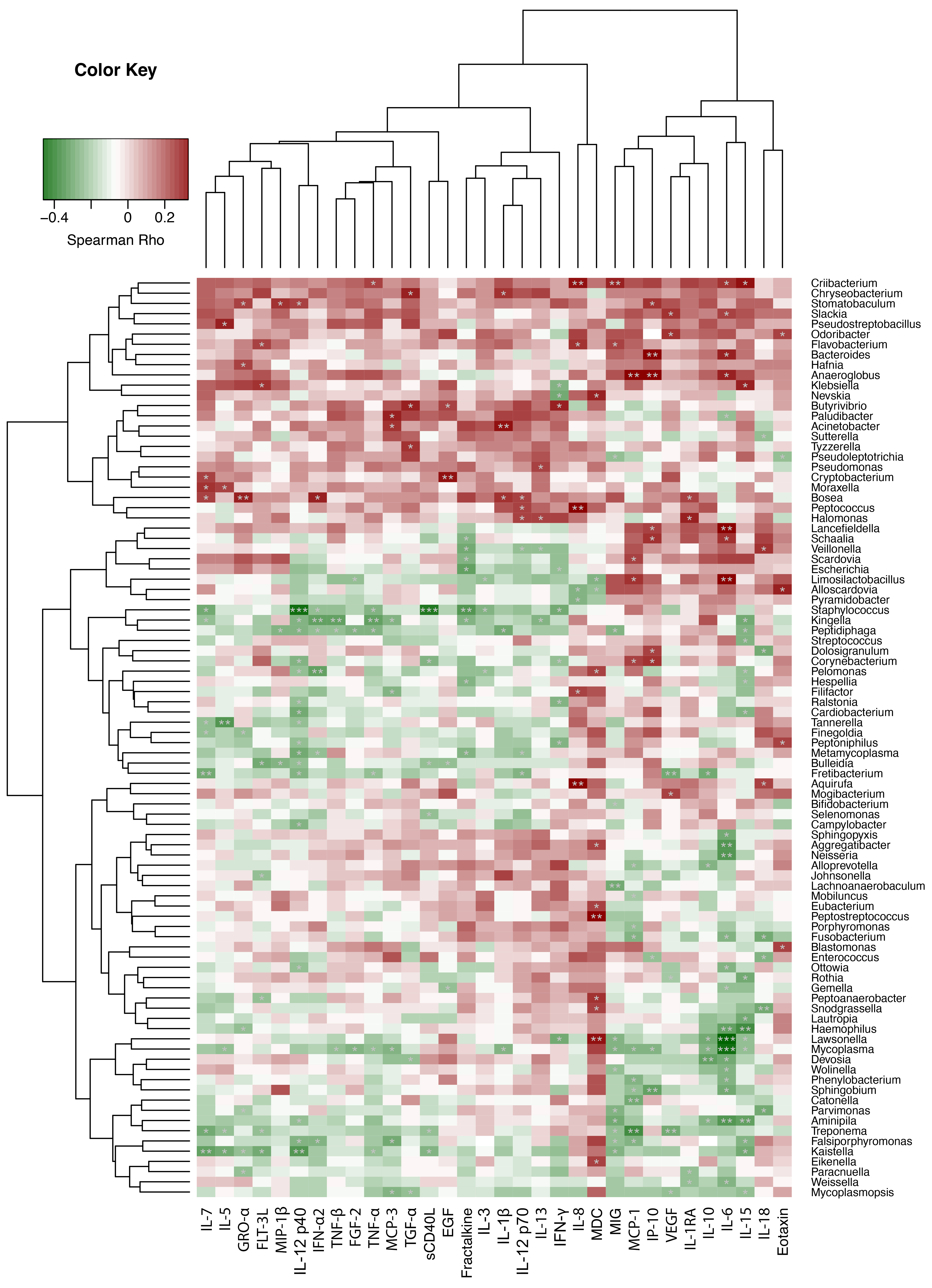

Supplement: Supplementary Figure 7 — Spearman correlation between bacterial genera and plasma cytokine in adult hospitalized COVID-19 patients (n = 90). [file Image_7.jpg]
